# First genetic report of *Ixodes kashmiricus* and associated *Rickettsia* sp*.*

**DOI:** 10.1186/s13071-022-05509-y

**Published:** 2022-10-19

**Authors:** Muhammad Numan, Nabeela Islam, Muhammad Adnan, Sher Zaman Safi, Lidia Chitimia-Dobler, Marcelo B. Labruna, Abid Ali

**Affiliations:** 1 Department of Zoology, Abdul Wali Khan University Mardan, Mardan, Khyber Pakhtunkhwa Pakistan; 2 Department of Chemistry, Abdul Wali Khan University Mardan, Mardan, Khyber Pakhtunkhwa Pakistan; 3 Department of Zoology, University of Peshawar, Peshawar, Khyber Pakhtunkhwa Pakistan; 4 Interdisciplinary Research Centre in Biomedical Materials, COMSATS University Islamabad Lahore Campus, Lahore, Punjab Pakistan; 5 Bundeswehr Institute of Microbiology, Munich, Germany; 6 Department of Preventive Veterinary Medicine and Animal Health, Faculty of Veterinary Medicine, University of São Paulo, São Paulo, Brazil

**Keywords:** Hard ticks, Transhumant herds, *Ixodes kashmiricus*, *Rickettsia*, Pakistan

## Abstract

**Background:**

Hard ticks (Ixodidae) are hematophagous ectoparasites that transmit various pathogens to a variety of hosts including humans. Transhumant herds have been involved in the spread of ticks and associated *Rickettsia* spp., and studies on this neglected topic have been unexplored in many regions including Pakistan. This study aimed to investigate ticks infesting transhumant herds of sheep (*Ovis aries*) and goats (*Capra hircus*) in district Shangla, Khyber Pakhtunkhwa, Pakistan.

**Methods:**

Of the 144 examined animals, 112 hosts (68 sheep and 44 goats) of transhumant herds were infested by 419 ticks of different life stages including nymphs (105; 25%), males (58; 14%) and females (256; 61%). For molecular analyses, DNA was extracted from 64 collected ticks and subjected to PCR for the amplification of tick 16S rDNA and ITS2 partial sequences and for the amplification of rickettsial *gltA* and *ompA* gene sequences.

**Results:**

All tick specimens were identified as *Ixodes kashmiricus* based on morphological features. The obtained 16S rDNA and ITS2 sequences showed 95.7% and 95.3% identity, respectively, with *Ixodes kazakstani* reported from Kyrgyzstan. In the phylogenetic tree, the sequences clustered with members of the *Ixodes ricinus* species complex, including *I. kazakstani* and *Ixodes apronophorus*. Additionally, rickettsial *gltA* and *ompA* partial sequences were 99.7% identical to *Rickettsia* sp. endosymbiont of *Ixodes* spp. from Panama and Costa Rica and 99.2% with *Rickettsia* endosymbiont from the USA. Phylogenetically, the rickettsial *gltA* and *ompA* partial sequences from *I. kashmiricus* clustered with various haplotypes of *Rickettsia* endosymbiont, which were sister cladded to *Rickettsia monacensis*.

**Conclusions:**

This is the first genetic report of *I. kashmiricus* and associated *Rickettsia* sp. Large-scale tick surveillance studies across the country are needed to investigate *Ixodes* ticks and associated pathogens.

**Graphical Abstract:**

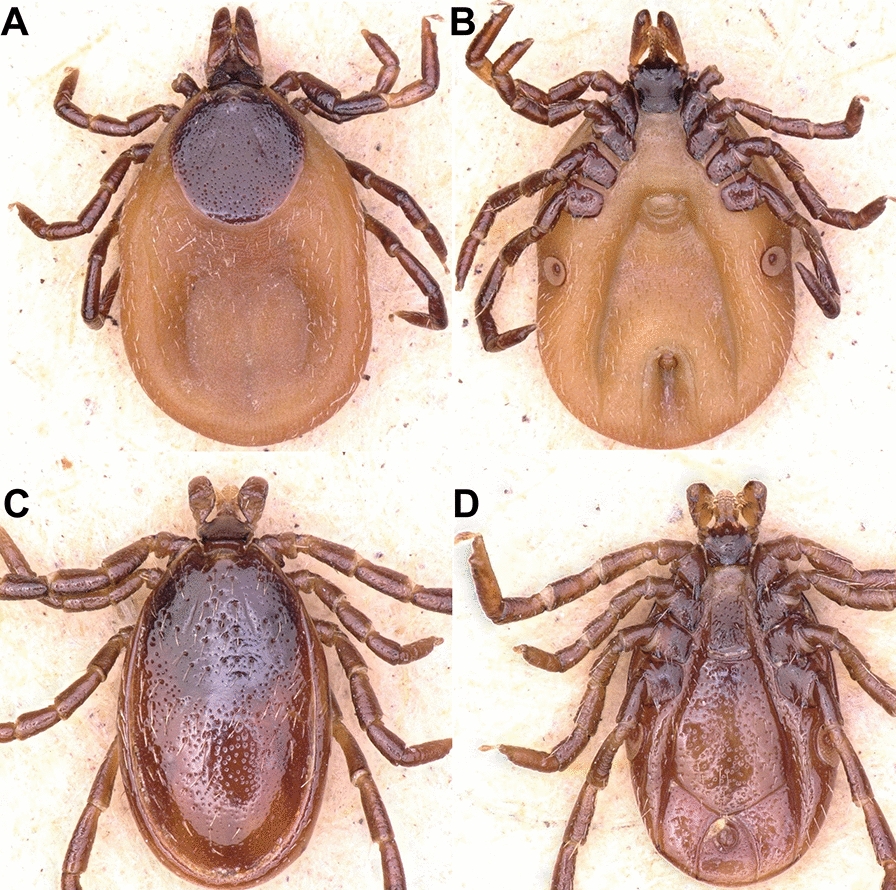

## Background

Hard ticks (Ixodida: Ixodidae) are hematophagous ectoparasites that adversely affect vertebrate hosts including amphibians, reptiles, birds and mammals, including humans [1–4]. Among ticks, several species spread and adopt to novel geographical regions with transhumant herds [5]. Globally, 18 subgenera comprised of 255 *Ixodes* species have been described [4, 6, 7]. Among them, subgenus *Ixodes* have closely related species, such as *Ixodes nipponensis, Ixodes kazakstani, Ixodes kashmiricus, Ixodes scapularis, Ixodes hyatti, Ixodes redikorzevi, Ixodes hymalayensis, Ixodes nuttallianus, Ixodes ricinus, Ixodes persulcatus, Ixodes pacificus, Ixodes pavlovskyi, Ixodes inopinatus, Ixodes minor* and *Ixodes granulatus*, included in the *I. ricinus* species complex distributed mostly in the Oriental, Nearctic and Palearctic regions [4, 7–11]. Evolutionarily, *Ixodes* ticks are considered a more basal lineage of the Ixodidae family [6].

*Ixodes* ticks mainly infest small ruminants, rodents, birds, carnivores and humans in cold deciduous, mixed forests and vegetative regions where rainfall is abundant and relatively more humid (> 80% relative humidity) [1, 6–15]. Environmental fluctuations, such as low temperature at high latitudes have been considered to limit the spread of *Ixodes* ticks and to a certain extent become a severe threat to novel hosts [4, 5, 16, 17].

A number of tick-borne rickettsial bacteria have been reported in *Ixodes* ticks: *Rickettsia helvetica, Rickettsia monacensis, Rickettsia japonica, Rickettsia sibirica*, *Rickettsia buchneri*, *Rickettsia cooleyi, “Candidatus* Rickettsia vini”, “*Candidatus* Rickettsia mendelii”, “*Candidatus* Rickettsia uralica”, “*Candidatus* Rickettsia thierseensis” and other *Rickettsia* spp. in different regions. Many of these *Rickettsia* spp. agents are probably endosymbionts of *Ixodes* spp., whereas a few have been associated with human rickettsiosis [2, 11, 18–26]. Most of the *Ixodes*-related *Rickettsia* spp. belong to more basal groups of rickettsiae, while a few are part of the Spotted Fever group [2].

Pakistan is an agricultural country where livestock has an important place in the national economy. The climate and geography of Pakistan offer suitable conditions for the establishment of a variety of tick species, likely increasing the risk of transmission of tick-borne pathogens [3, 27–29]. The tick fauna of Pakistan includes species of the argasid genera *Argas*, *Carios* and *Ornithodoros*, the ixodid Metastriata genera *Rhipicephalus, Haemaphysalis*, *Hyalomma*, *Amblyomma* and *Nosomma*, and the ixodid Prostriata genus *Ixodes* [1, 3, 27, 28, 30–34]. For the latter genus, only two species, *Ixodes hyatti* and *Ixodes redikorzevi*, have been identified in Pakistan, both based only on morphology [35–37]. Studies on *Rickettsia* spp. associated with Prostriata ticks infesting transhumant herds have been neglected in the country.

In this study, we report *I. kashmiricus* ticks infesting transhumant herds (sheep, *Ovis aries*; goats, *Capra hircus*) and screened the collected ticks for *Rickettsia* spp. in Shangla district, Khyber Pakhtunkhwa (KP), Pakistan. Genetic characterization of an *Ixodes* species from Pakistan was performed for the first time to our knowledge.

## Methods

### Study area

**Fig. 1:**
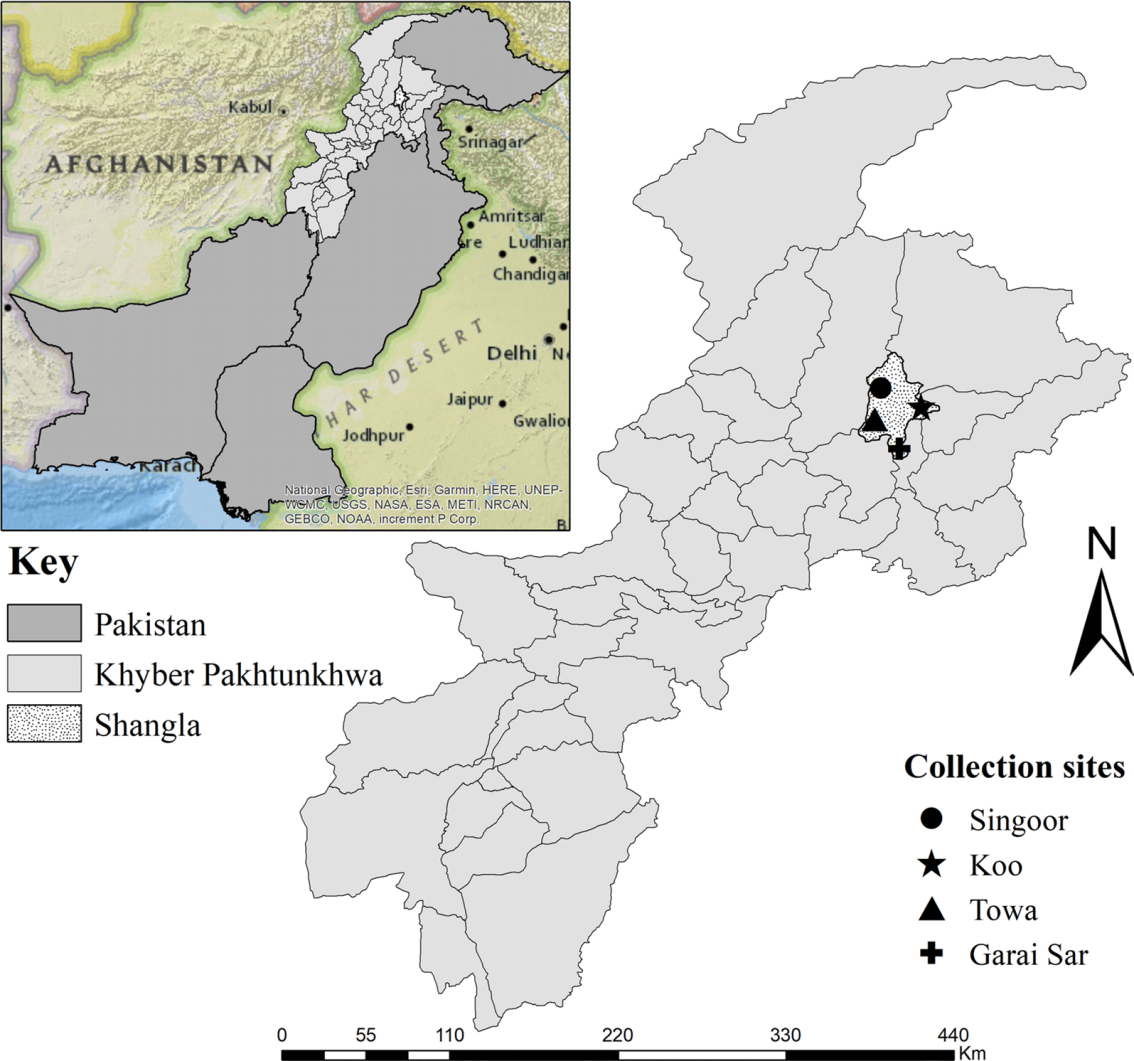
Map showing the collection sites of *Ixodes* species in Shangla district, Khyber Pakhtunkhwa (KP), Pakistan

Shangla district (34°47′32.1"N, 72°41′26.4"E) is situated 331 km northwest of Islamabad, the capital of Pakistan, covering 1586 km^2^. Shangla is surrounded by the Kohistan district to the north, Torghar and Battagram to the east, Swat to the west and Buner to the south. This is a hilly and mountainous region, with an elevation of 3440 m above sea level. Temperature ranges between 17 °C and 30 °C in summer and − 5 °C to 10 °C in winter and annual precipitation remains above 1200 mm (worldweatheronline.com). Ticks were collected from four mountainous regions in two tehsils (subdivision of a district), including Puran (Singoor, Towa and Garai Sar) and Chakesar (Koo) in Shangla district. “Google Earth Pro v 7.3” was used to find the exact geographic coordinates of collection sites, and the study map was designed via ArcGIS v 10.3.1 (Fig. 1).

### Tick collection and preservation

Tick samples were collected from transhumant herds of sheep and goats from February 2019 to November 2021. Tweezers were used to safely remove tick species without any damage. Relevant information such as date of collection, location, temperature, humidity and geo-coordinates of collection points were noted. The collected specimens were cleaned with distilled water and preserved in 100% ethanol.

### Morphological identification

**Fig. 2:**
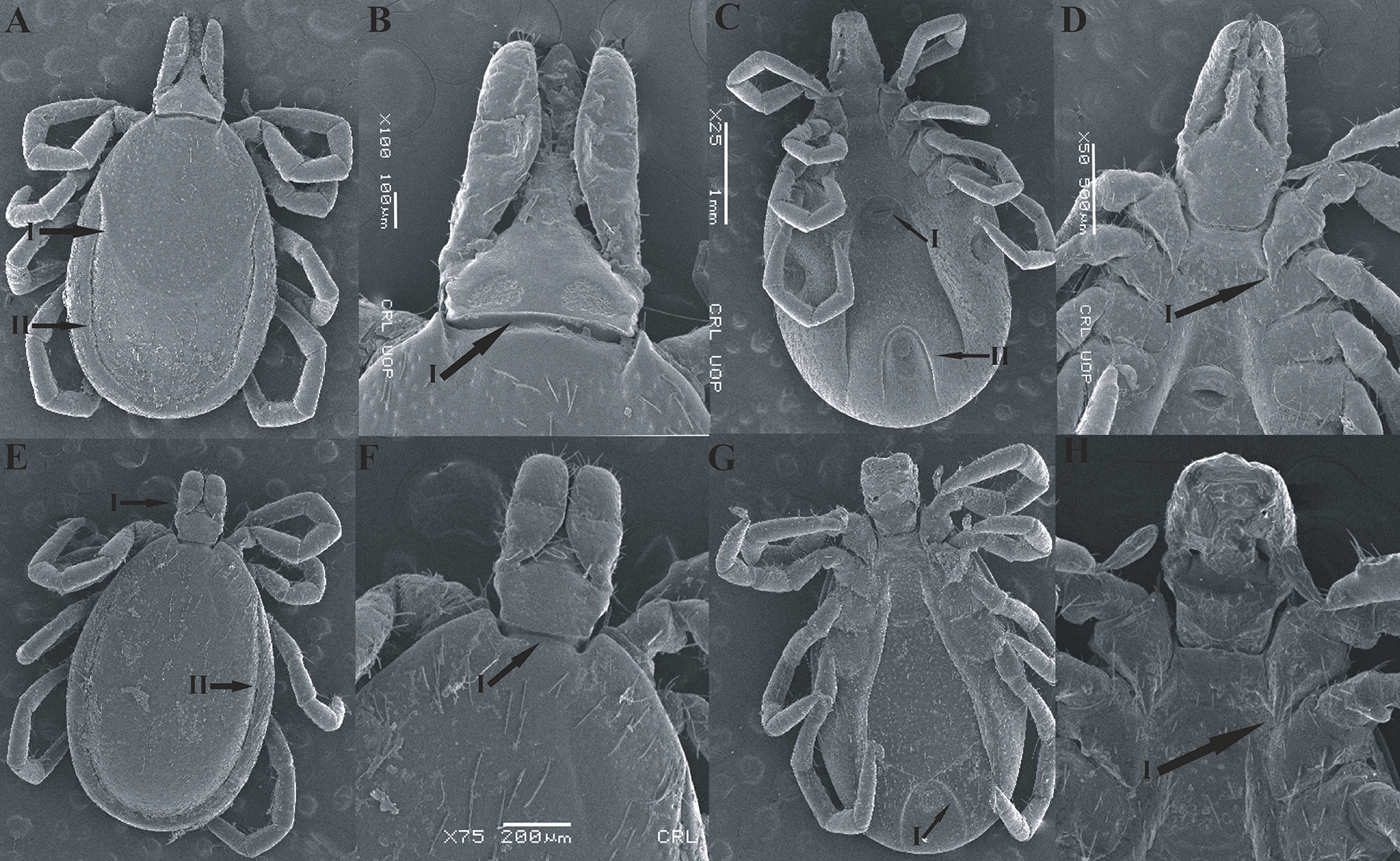
Female (upper row) and male (lower row) of *Ixodes kashmiricus*. **A**: female dorsal (I: scutal margin, II: marginal groove), **B**: female dorsal (I: posterior margin of basis capituli), **C**: female ventral (I: genital aperture, II: anal groove), **D**: female ventral (I: coxa I internal spur), **E**: male dorsal (I: capitulum, II: marginal groove), **F**: male dorsal (I: posterior margin of basis capituli), **G**: male ventral (I: anal groove), **H**: male ventral (I: coxa I internal spur)

Collected ticks were identified by using a Keyence microscope (Illinois, VHX 900F, USA, Itasca) with 50–200× magnification following the current literature [8, 38]. The specimens were photographed via scanning electron microscope (JSM5910, JEOL, Japan) in Centralized Resource Laboratory (CRL) at the University of Peshawar (Fig. 2).

### DNA extraction and PCR

A total of 64 tick specimens (32 nymphs, 17 males and 15 females) were selected randomly from hosts of all regions and were individually subjected to the extraction of genomic DNA by using the phenol-chloroform method [39]. The concentration of the extracted DNA samples was quantified by using Nanodrop (Nano-Q, Optizen, Daejeon, South Korea).

The extracted DNA was used for the amplification of fragments of the mitochondrial 16S ribosomal RNA gene and the nuclear second internal transcribed spacer (ITS2). Conventional PCR assays were performed in microtubes containing 20 µl reaction mixture containing: 1 µl (10 µM) of each (forward and reverse) primer (Table 1), 2 µl DNA (50–100 ng/µl), 4 µl PCR water (nuclease free) and 12 µl Dream*Taq* Green MasterMix (2X) (Thermo Fisher Scientific, Inc., Waltham, MA, USA). Conditions for thermocycling were: initial denaturation was 95 °C for 3 min, followed by 35 cycles of 95 °C for 30 s, 55 °C for 30 s (16S rRNA), 55 °C for 60 s (ITS2) and extension 72 °C for 30 s and final extension at 72 °C for 7 min. The PCR contained negative (PCR water) and positive (*Rhipicephalus microplus* DNA) control. The PCR products were run in a 2% agarose gel, dyed with 4 µl ethidium bromide and observed in Gel Documentation System (BioDoc-It™ Imaging Systems UVP, LLC, Upland, CA, USA).

**Table 1:** List of primers used for the amplification of targeted gene sequences of *Ixodes* tick and associated rickettsial species

Organism/gene	PCR	Primer	Sequence 5' to 3'	Amplicon size	References
Tick/16S rRNA	PCR	16S + 1	CCGGTCTGAACTCAGATCAAGT	460 bp	[40]
		16S-1	GCTCAATGATTTTTTAAATTGCTG		
Tick/ITS2	PCR	5.8S	CCATCGATGTGAAYTGCAGGACA	800 bp	[41]
		28S	GTGAATTCTATGCTTAAATTCAGGGGT		
*Rickettsia* spp./*gltA*	RT-PCR	CS-5	GAGAGAAAATTATATCCAAATGTTGAT	147 bp	[42]
		CS-6	AGGGTCTTCGTGCATTTCTT		
*Rickettsia* spp./*gltA*	PCR	CS-78	GCAAGTATCGGTGAGGATGTAAT	401 bp	[42]
		CS-323	GCTTCCTAAAATTCAATAAATCAGGAT		
*Rickettsia* spp./*ompA*	PCR	Rr190.70	ATGGCGAATATTTCTCCAAAA	631 bp	[43]
		Rr190.701	GTTCCGTTAATGGCAGCATCT		

### Screening for rickettsial DNA

Extracted DNA was used for the detection of rickettsial DNA by the amplification of a short fragment of the citrate synthase (*gltA*) gene using specific primers (Table 1) in a real-time PCR (Applied Biosystems, Thermo Fisher Scientific, Waltham, MA, USA) [42]. Positive specimens for rickettsial DNA were further screened by the amplification of a fragment of the rickettsial 190-kDa outer membrane protein (*ompA*) and a larger fragment of the rickettsial *gltA* gene using specific primers (Table 1) in conventional PCR assays (BIOER, China). PCR reaction mixture of 20 µl contained 1 µl (10 µM) of each primer (Table 1), 2 µl extracted DNA (50–100 ng/µl), 4 µl PCR water and 12 µl Dream*Taq* Green MasterMix (2×) (Thermo Fisher Scientific, Inc., Waltham, MA, USA). Cycling conditions for the amplification of rickettsial DNA were: initial denaturation at 95 °C for 3 min, followed by 40 cycles at 95 °C for 20 s, 56 °C for 30 s (*gltA*), 55 °C for 30 s (*ompA*), extension at 72 °C for 30 s and final extension at 72 °C for 7 min. In each PCR reaction, PCR water and *Rickettsia massiliae* DNA were used as a negative and positive control, respectively. PCR products were electrophoresed in 2% agarose gel, and the results were visualized under ultraviolet light using a Gel Documentation System (UVP BioDoc-It Imaging system, UVP, LLC, Upland, CA, USA).

All PCR-positive products of ticks (16S rRNA and ITS2) and associated *Rickettsia* (*gltA* and *ompA*) were purified by ExoSAP-IT Kit (Thermo Fisher Scientific, Waltham, MA, USA) following the manufacturer’s protocol. Purified PCR products were sent for DNA sequencing bidirectionally (Macrogen Inc., Seoul, South Korea) using the same primers used in the PCR amplification.

### DNA sequencing and phylogenetic analyses

The obtained sequences were trimmed to remove primer sequences and contaminated and poor-quality nucleotides in the flanks by using SeqMan V 5.0 (DNASTAR Inc., Madison, WI, USA). The purified or cropped sequences were subjected to a Basic Local Alignment Search Tool (BLAST) [44] at the National Center for Biotechnology Information (NCBI). Homologous sequences along with ancestor species as an outgroup were downloaded in FASTA format based on their high percentage identity. The downloaded sequences were subjected to ClustalW Multiple alignment [45] in BioEdit Sequence Alignment Editor V 7.0.5 (Raleigh, NC, USA) [46]. The phylogenetic tree was constructed separately for tick sequences (16S rDNA and ITS2) and *Rickettsia* sequences (*gltA* and *ompA*) by Molecular Evolutionary Genetics Analysis (MEGA-X), aligned by the MUSCLE algorithm [47], and used the maximum likelihood method based on the Tamura-Nei model with bootstrapping value at 1000 replications [48].

## Results

### Tick morphological identification

A total of 419 hard ticks (Table 2) were collected and morphologically identified as *I. kashmiricus*, a member of the *I. ricinus* species complex. During collection, no other tick species were found infesting the small ruminants.

**Table 2:** Tick identified as *Ixodes kashmiricus* in different geographical location in Pakistan, hosts and tick life stages, and detection of *Rickettsia* sp. DNA in the ticks

Location	Hosts	Number examined animals	Number infested animals (%)	Number of collected ticks (N: nymphs, M: males; F: females)	Number of ticks tested by molecular analyses	Amplified *gltA* and *ompA* for *Rickettsia*
Singoor	Sheep	21	17	80 (21N, 9M, 50F)	9 (4 N, 3 M, 2F)	7 (4 N, 1 M, 2F)
	Goats	17	10	25 (5N, 3F, 17M)	4 (2N, 1M, 1F)	3 (1N, 1M, 1F)
Koo	Sheep	19	18	111 (28N, 15M, 68F)	11 (6N, 3M, 2F)	8 (5N, 1M, 2F)
	Goats	18	12	27 (8N, 7M, 12F)	5 (3N, 1M, 1F)	3 (1N, 1M, 1F)
Towa	Sheep	14	14	47 (11N, 6M, 30F)	8 (5N, 2M, 1F)	8 (5N, 2M, 1F)
	Goats	16	10	24 (6N, 4M, 14F)	5 (2N, 1M, 2F)	4 (1N, 1M, 2F)
Garai Sar	Sheep	20	19	86 (19N, 11M, 56F)	16 (7N, 4M, 5F)	12 (4N, 3M, 5F)
	Goats	19	12	19 (7N, 3M, 9F)	6 (3N, 2M, 1F)	3 (1N, 1M, 1F)
Total sheep		74	68 (92)	324 (79N, 41M, 204F)	44 (22N, 12M, 10F)	35 (80%) (18N, 7M, 10F)
Total goats		70	44 (63)	95 (26N, 17M, 52F)	20 (10N, 5M, 5F)	13 (65%) (4N, 4M, 5F)
Overall total		144	112 (78)	419 (105N, 58M, 256F)	64 (32N, 17M, 15F)	48 (75%) (22N, 11M, 15F)

Briefly, morphological identification of adult female ticks relies on a shorter inner spur on coxa I; all coxae have a short external spur; scutum is covered with uniform, larger punctuations; setae of the scutum are sparse. Scapulae have the shape of slightly long or pointed teeth. The posterior valve is convex. The stigmas are irregularly oval and elongated (Fig. 2A–D).

Males lack auriculae, idiosoma broadly oval, the posterior pair of teeth of the hypostome is relatively short and poorly developed, and the posterior margin of the basis capituli forms a rounded line on the ventral side. Punctuations on the conscutum are smoothed, larger, deepened punctuations. Conscutum is flattened with long setae; the longest setae are located on the lateral parts of the conscutum and on the marginal carina. Scapulae are small and lateral carinae absent. The stigmas are relatively small, roundish or oval, elongated in the longitudinal direction (Fig. 2E–H).

Nymphs contained a broadly oval, rounded scutum with several setae, posterior margin slightly kinked; alloscutum setae longer than those of the scutum. Hypostome widest at base, wedge-shaped, narrowed, apex rounded; shorter than in *I. pavlovskyi*, but longer than in *I. kazakstani*, which are the main features of *I. kashmiricus*.

### Hosts

Overall, 144 small ruminants (74 sheep and 70 goats) were examined in four villages (38 animals in Singoor, 37 in Koo, 30 in Towa and 39 in Garai Sar) of Shangla district (Table 2). Of them, 112 (78%) hosts including 68 (92%) sheep and 44 (63%) goats were infested by 419 *I. kashmiricus* of different life stages. Among them, sheep (68 out of 74 hosts) were more infested, with a significantly higher (*P* < 0.01) prevalence. Moreover, the number of infested sheep was higher than infested goats in each of the four sampled locations (Singoor, Koo, Towa, Garai Sar) (Table 2).

### Molecular analyses of ticks

A total of 64 tick specimens were tested by molecular analyses and generated amplicons of the expected size through PCR assays targeting the 16S rRNA and ITS2 genes. Partial sequences of the 16S rDNA mitochondrial gene were generated for 31 nymphs, 16 males and 12 females, which were identical to each other; the remaining PCR-positive ticks (1 nymph, 1 male, 3 females) did not generate high-quality sequences. By BLAST analysis, the partial 16S rDNA sequence (415 bp) of *I. kashmiricus* from Pakistan was most similar (95.7%) to the sequences of *I. kazakstani* from Kyrgyzstan (MF095803-MF095806) and then 94.7% similar to several sequences of *Ixodes apronophorus* from Russia (MH790193-MH790198). A consensus partial sequence of the ITS2 nuclear gene (754 bp) was generated for five ticks, which by BLAST analysis was the most similar (95.3%) to the sequences of *I. kazakstani* from Kyrgyzstan (MF095819-MF095822) and then 85.3–85.8% to some sequences of *I. apronophorus* reported from Russia (MH784894-MH784898).

Among the 64 ticks tested by molecular analyses, rickettsial DNA was detected in 48 (75%) ticks (22 nymphs, 11 males, 15 females), including specimens collected from sheep or goats and from each of the four locations (Table 2). These ticks generated identical *gltA* sequences, which by BLAST analysis were most similar (99.7%; 349/350 bp) to *Rickettsia* sp. endosymbiont of *Ixodes boliviensis* from Panama (MW699695) and Costa Rica (KU529481) and *Ixodes tapirus* from Panama (MW699691). The same tick specimens generated identical *ompA* sequences, which by BLAST analysis were the most similar (99.2%; 366/369 bp) to *Rickettsia* sp. endosymbiont of *Ixodes pacificus* (KX505847) from the USA.

### Phylogenetic analyses

A total of 27 and 30 partial sequences of the 16S rDNA and ITS2, respectively, of *Ixodes* spp. were downloaded from GenBank. In the case of *Rickettsia* spp., 30 and 33 sequences of *gltA* and *ompA* genes, respectively, were downloaded and used in the alignments.

**Fig. 3:**
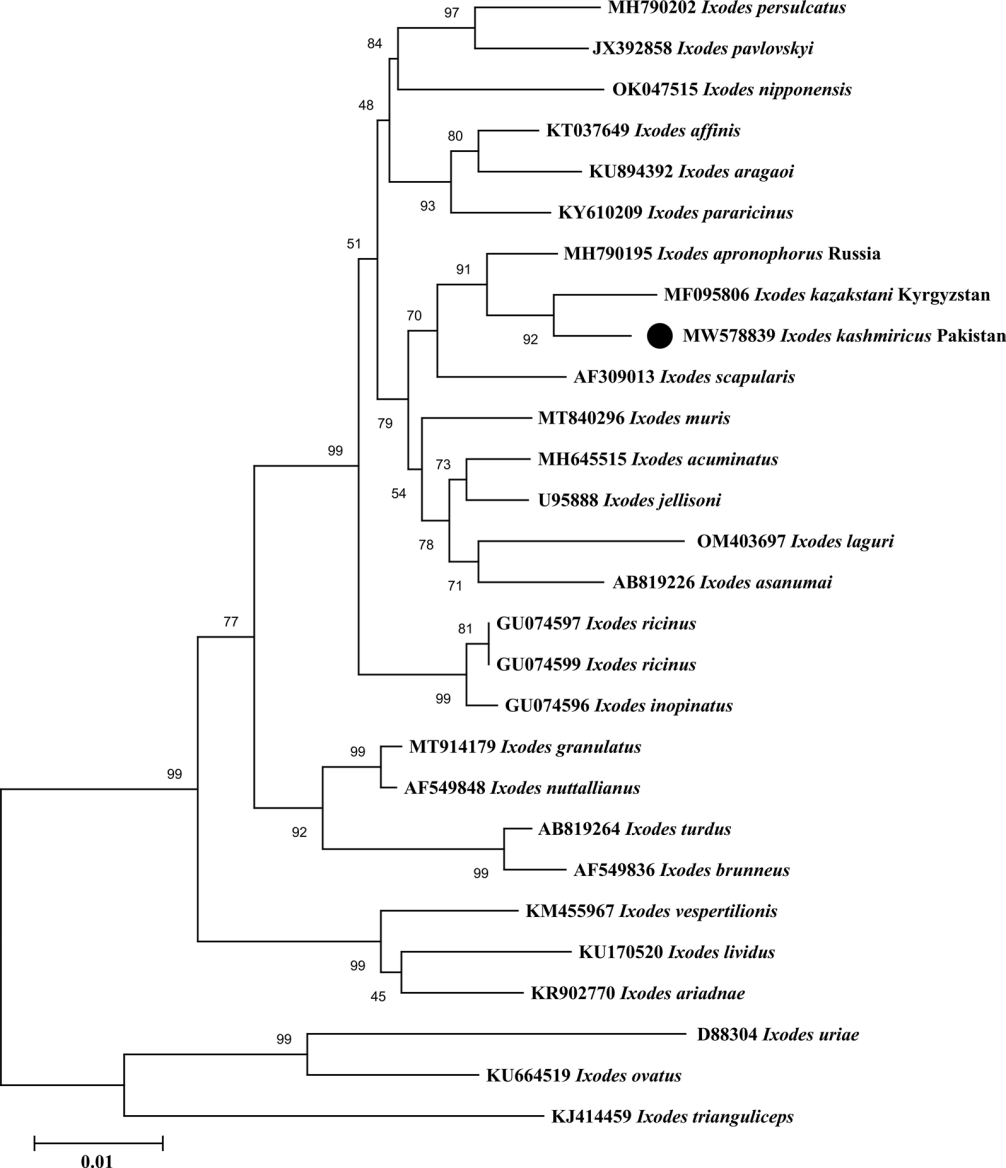
Maximum likelihood phylogenetic tree based on 16S rDNA partial sequence of *Ixodes kashmiricus*. The *Ixodes trianguliceps*, *Ixodes uriae* and *Ixodes ovatus* sequences were taken as an outgroup. The levels of bootstrap support (> 50%) for phylogenetic groupings are given at each node; the accession numbers are followed by the species name and location. The obtained sequence was represented by black circle (MW578839)

**Fig. 4:**
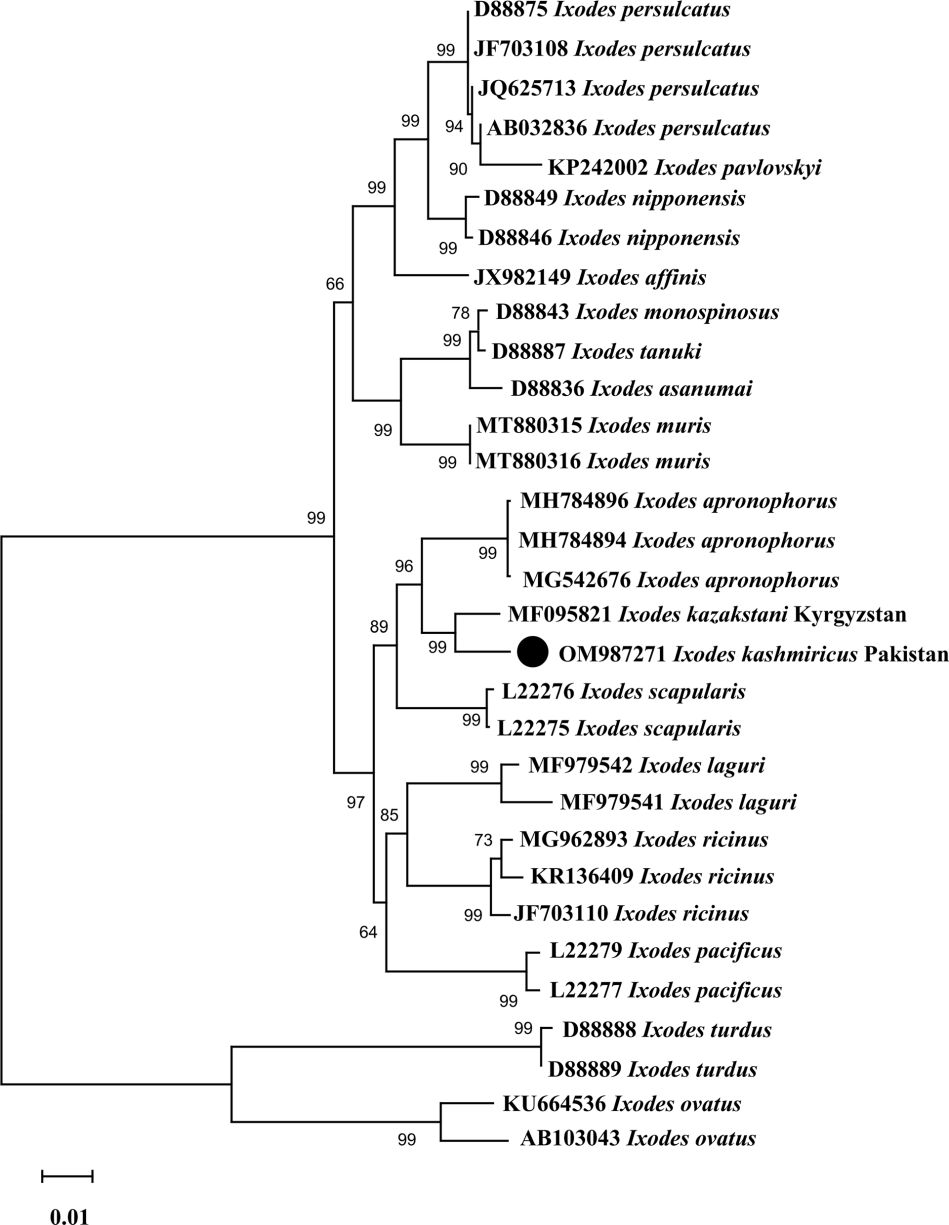
Maximum Likelihood phylogenetic tree based on ITS2 partial sequence of *Ixodes kashmiricus*. The *Ixodes turdus* and *Ixodes ovatus* sequences were taken as an outgroup. The levels of bootstrap support (> 50%) for phylogenetic groupings are given at each node; the accession numbers are followed by the species name and location. The obtained sequence was represented by black circle (OM987271)

In the phylogenetic tree inferred from partial sequences of the 16S rDNA gene, *I. kashmiricus* from Pakistan (MW578839) clustered with *I. kazakstani* (MF095806) and *I. apronophorus* (MH790195) reported from Kyrgyzstan and Russia, respectively (Fig. 3). Similarly, in the ITS2 phylogenetic tree, the partial sequence of Pakistan (OM987271) clustered again with *I. kazakstani* (MF095821) and *I. apronophorus* (MH784894, MH784896 and MG542676) reported from Kyrgyzstan and Russia, respectively (Fig. 4). In both phylogenetic trees, the 16S rDNA and ITS2 partial sequences clustered within a large clade composed by *Ixodes* species of the *I. ricinus* species complex.

**Fig. 5:**
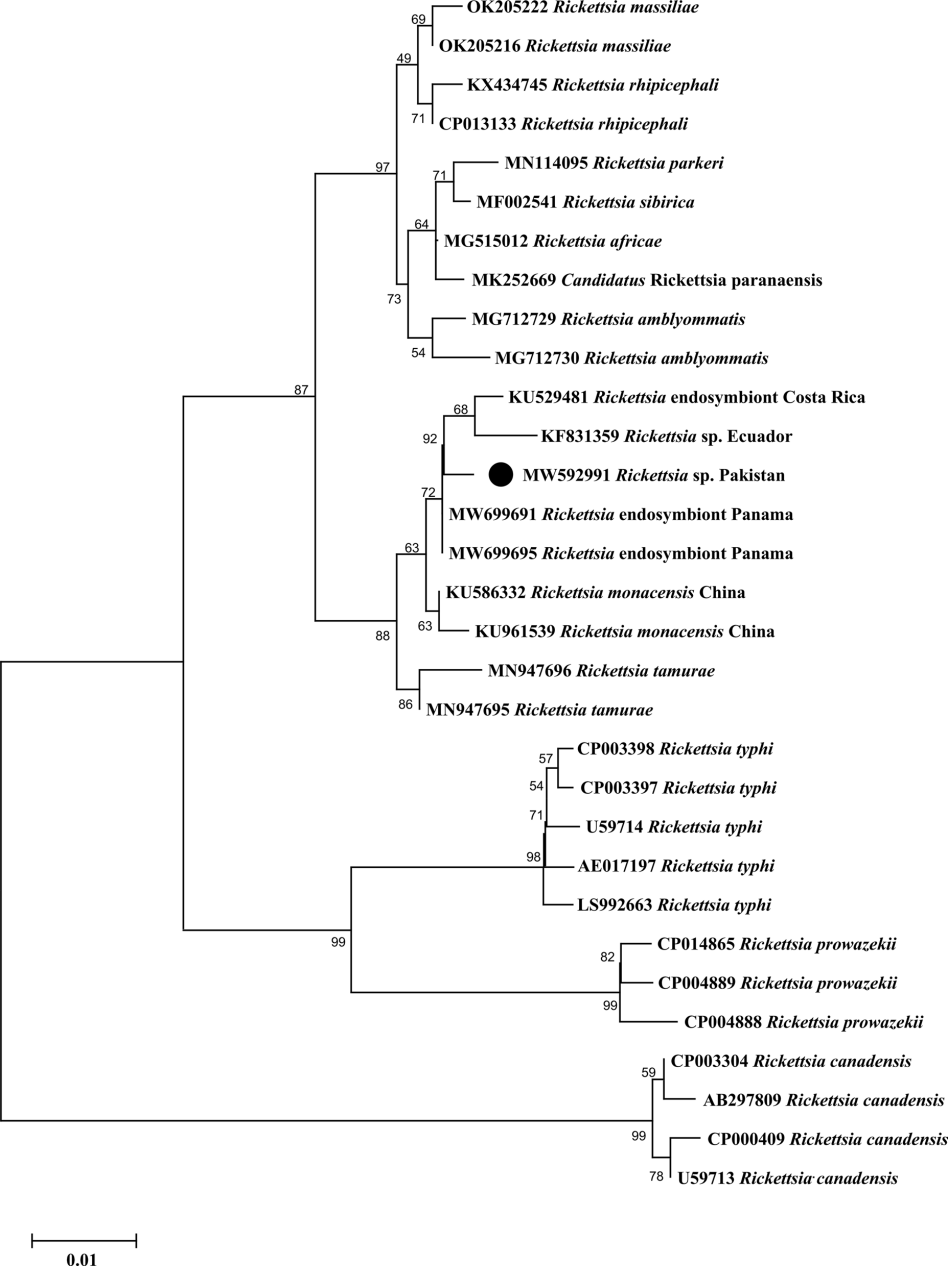
Maximum likelihood phylogenetic tree based on *gltA* sequence for *Rickettsia* sp. from *Ixodes kashmiricus*. The *Rickettsia canadensis* sequences were taken as an outgroup. The levels of bootstrap support (> 50%) for phylogenetic groupings are given at each node; the accession numbers are followed by the species name and location. The obtained sequence was represented by a black circle (MW592991)

**Fig. 6:**
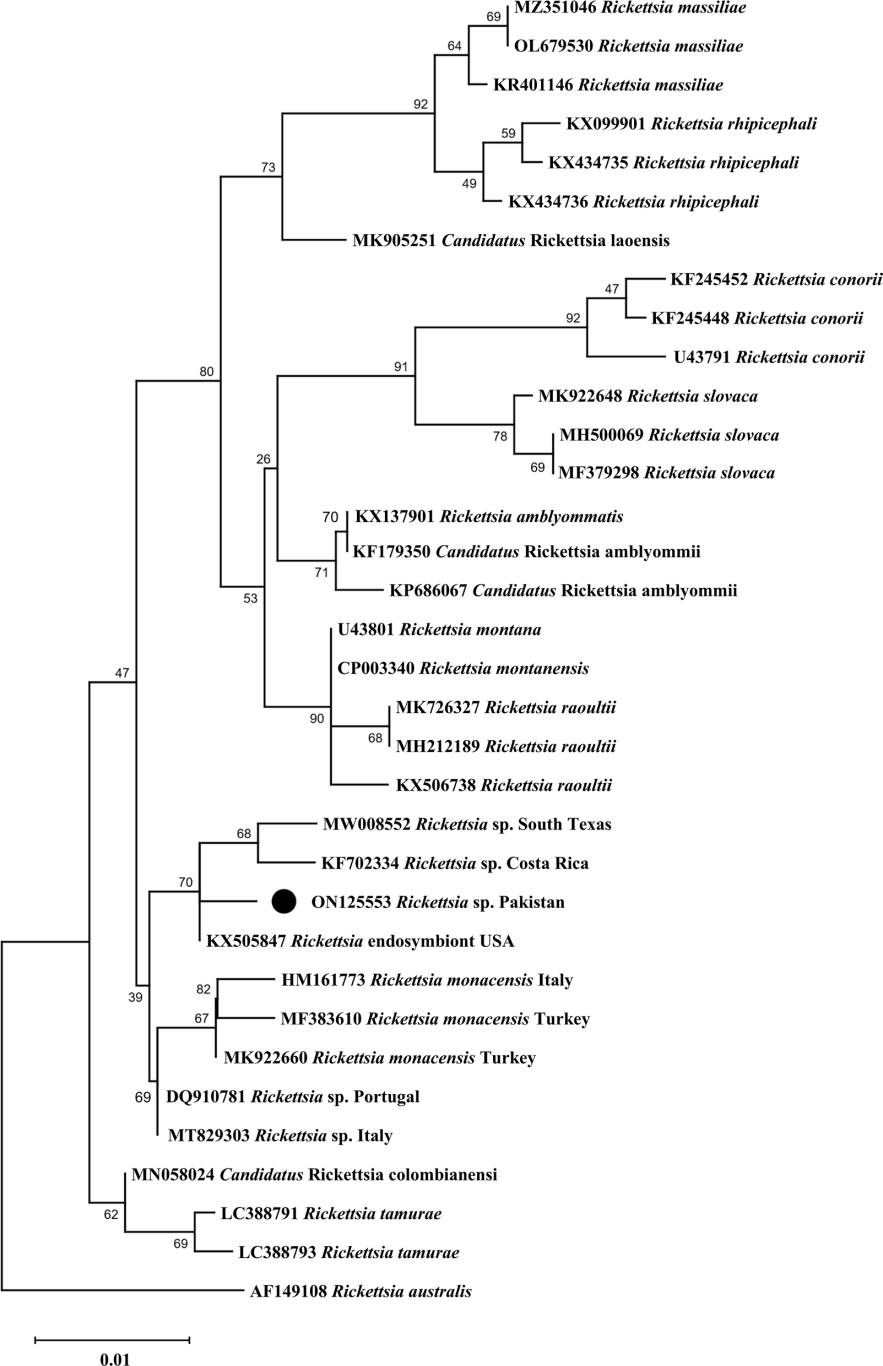
Maximum likelihood phylogenetic tree based on *ompA* sequence of *Rickettsia* sp. from *Ixodes kashmiricus*. The *Rickettsia australis* sequence was taken as an outgroup. The levels of bootstrap support (> 50%) for phylogenetic groupings are given at each node; the accession numbers are followed by the species name and location. The obtained sequence was represented by a black circle (ON125553)

In the phylogenetic trees inferred from partial sequences of the rickettsial genes, the *gltA* (MW592991) and *ompA* (ON125553) partial sequences from *I. kashmiricus* in Pakistan formed a clade with different haplotypes of *Rickettsia* endosymbionts of *Ixodes* spp. from Costa Rica, Panama and the USA, which were sister to *R. monacensis*, a species associated primarily with *I. ricinus* (Figs. 5 and 6). These sequences were sister or basal to the *Rickettsia* species typically belonging to the spotted fever group.

## Discussion

*Ixodes* ticks have been observed as vector reservoirs for tick-borne relapsing fever, tick-borne encephalitis, Lyme borreliosis and babesiosis [2, 11, 21, 49]. The diversity of *Ixodes* ticks reported in various regions is still unexplored in Pakistan. In Pakistan, the local population faces large economic and health problems due to the lack of knowledge about ticks and associated disease-causing agents. Based on morphology, *I. hyatti* from Peshawar and *I. redikorzevi* from Kaghan and an *Ixodes* sp. identified at genus level from Swat have been reported in Pakistan [35–37]. Until the present study, *Ixodes* species had never been genetically identified in Pakistan. In this study, ticks collected from transhumant herds in district Shangla were morphologically and genetically characterized as *I. kashmiricus* and associated *Rickettsia* sp. for the first time to our knowledge.

During this study, large ruminants such as cattle, Asian water buffaloes and equids were also observed (data not shown); however, *I. kashmiricus* was found infesting only small ruminants (sheep and goats) of transhumant herds. Among the small ruminants, the significantly high infestation on sheep suggests the preference of this tick to sheep as a suitable host. Many *Ixodes* spp. of the *I. ricinus* species complex are associated with small ruminants [1, 8, 10], which might graze in areas with favorable environmental conditions for the survival of ticks [6, 13]. Likewise, the district Shangla is a hilly and high mountainous region having mild summer (17 °C to 30 °C), cold winter (− 5 °C to 10 °C), relative humidity approximately 80%, heavy rainfall in all seasons (spring, summer, autumn and winter) and annual precipitation above 1200 mm. It is important to mention that these transhumant herds annually migrate to northern regions between April and September at the frontiers of the country in Gilgit Baltistan, Kohistan (Dassu) and Chitral. These regions share borders with Afghanistan to the north, China to the northeast, India (Jammu and Kashmir) to the southeast and AJK (Azad Jammu and Kashmir) to the south of Pakistan. Moreover, these boundaries have been linked to the former Union of Soviet Socialist Republics (USSR). The USSR countries have been associated with the distribution of some ancestral *Ixodes* species because of the occurrence of favorable environmental conditions [8, 9].

To date, *Ixodes* ticks have not been genetically characterized from Pakistan. The genetic identification of the collected ticks validated the morphological compatibility, as the 16S rDNA and ITS2 sequences clustered in a phylogenetic tree with the sequences of *I. kazakstani* and *I. apronophorus* (*I. ricinus* species complex) reported from Kyrgyzstan and Russia, respectively. The topology of the phylogenetic trees of *I. kashmiricus* were compared with the species of the *I. ricinus* group [7, 50]. The phylogenetic tree based on the 16S rDNA and ITS2 partial sequences were congruent and confirmed that *I. kashmiricus* belongs to the *I. ricinus* species complex. In the pairwise alignment, the 16S rDNA and ITS2 partial sequences of *I. kashmiricus* showed a minimum nucleotide difference of 18 and 27 bp, respectively, which showed 5% genetic difference with *I. kazakstani* followed by *I. apronophorus* of the *I. ricinus* species complex.

*Ixodes kashmiricus* was described by Pomerantzev in 1948 and has been reported only from India-Jammu and Kashmir, Vardvan-Maru River and the northern stream of Chenab River region in India [8, 10]. This location is ≈300 km away from the present locality in Pakistan. Until the present study, there were no molecular genetic data or DNA sequence for *I. kashmiricus*. Herein, we genetically characterized *I. kashmiricus* based on 16S rDNA and ITS2 partial sequences for the first time to our knowledge; they shared high identity with two available sequences of *I. ricinus* species complex—*I. kazakstani* and *I. apronophorus.*

*Ixodes* ticks are commonly reported as infected by rickettsial endosymbionts, although a few *Ixodes* species have also been implicated as a vector of rickettsial agents, like for example *R. australis, R. monacensis* and *R. helvetica* [2, 18, 23, 43]. The present study reported *Rickettsia* sp. in *I. kashmiricus* that grouped with *Rickettsia* endosymbionts of *Ixodes* spp. of the *I. ricinus* species complex. The pathogenicity of the *Rickettsia* sp. detected in this study remains to be investigated given the importance of this bacterial group as an agent of emerging infectious tick-borne diseases. However, the high infection rate (75%) of *Rickettsia* sp. in *I. kashmiricus* is compatible with an endosymbiont.

## Conclusions

This study genetically characterized *I. kashmiricus* and associated *Rickettsia* sp. for the first time to our knowledge. Morphological and phylogenetic analyses of the collected ticks showed close resemblance to *I. kashmiricus* and clustered with members of the *I. ricinus* species complex, including *I. kazakstani* and *I. apronophorus*. A *Rickettsia* sp. was detected in *I. kashmiricus* and shown to be genetically related to *Rickettsia* sp. endosymbiont of other *Ixodes* spp. of the *I. ricinus* species complex. These results may assist our understanding of the epidemiology of *Ixodes* ticks and associated *Rickettsia* sp. and reinforce country-wide tick surveillance programs.

## Data Availability

The datasets to support the conclusions of this article are given within the article.

## Abbreviations

BLAST: Basic Local Alignment Search Tool
CRL: Centralized Resource Laboratory
*gltA*: Citrate synthase
ITS2: Internal transcribed spacer 2
KP: Khyber Pakhtunkhwa
MEGA: Molecular evolutionary genetics analysis
NCBI: National Center for Biotechnology Information
*ompA*: Outer membrane protein
PCR: Polymerase chain reaction
rDNA: Ribosomal DNA
